# Adsorption and Decomposition Mechanisms of Li_2_S on 2D Thgraphene Modulated by Doping and External Electrical Field

**DOI:** 10.3390/ma18143269

**Published:** 2025-07-10

**Authors:** Ruofeng Zhang, Jiyuan Guo, Lanqing Chen, Fengjie Tao

**Affiliations:** School of Science, Jiangsu University of Science and Technology, Zhenjiang 212100, China; zhangruofeng621@stu.just.edu.cn (R.Z.);

**Keywords:** lithium–sulfur batteries, first-principles calculation, thgraphene, doping, external electric field

## Abstract

The modification of materials is considered as one of the productive methods to facilitate the better electrochemical behavior of lithium–sulfur battery cathodes and inhibit the shuttle effect. Adopting first-principles calculations in this work, the application potential of pristine and B-, N-, and P-doped thgraphene as anchoring materials was investigated. The results reveal that pristine and doped substrates have an excellent structural stability, conductivity, and electrochemical activity. In the absence of an electric field, four substrates exhibit a strong anchoring effect on the Li_2_S cluster, where the adsorption energies fall within 3.10 to 4.48 eV. Even under the external electric field, all substrates exhibit notable structural stability during Li_2_S adsorption processes and maintain a high electrical conductivity, with adsorption energies exceeding 2.75 eV. Furthermore, it has been observed that the interfacial diffusion energy barriers for Li on all substrates are below 0.35 eV, which effectively enhances Li migration and facilitates reaction kinetics. Additionally, Li_2_S demonstrates a low decomposition energy barrier (varying from 0.84 to 1.55 eV) on pristine and doped substrates, enabling the efficient regeneration of the active material during the battery cycling. These findings offer a scientific guideline for the design of pristine and doped thgraphene as an excellent anchoring material for advanced lithium–sulfur batteries.

## 1. Introduction

**Figure 1:**
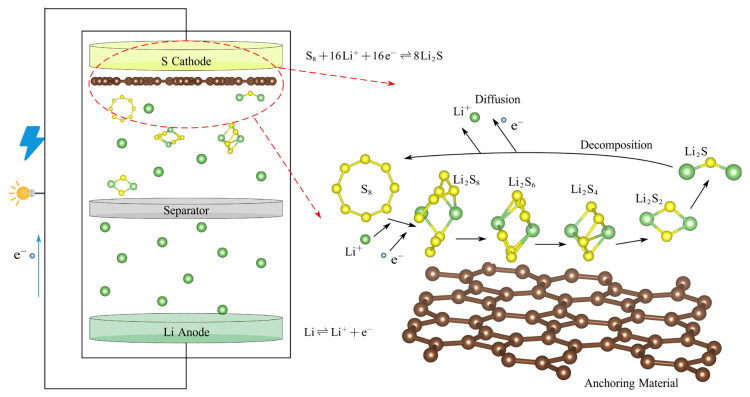
The schematic diagram of the structure of a lithium–sulfur battery.

Given the steady growth in energy consumption, the world is witnessing a rising global demand for sustainable and efficient energy storage solutions [1,2,3]. Concerning the storage of electrical energy, advanced electrochemical energy storage systems [4,5] with efficient energy conversion characteristics, environmental friendliness, and recyclability are crucial in improving energy utilization efficiency [6]. Over the past few years, lithium–sulfur batteries (LiSBs) [7,8] have become a key competitor in aerospace satellites, marine exploration, portable electronic devices, etc., with an impressive energy density of 2600 Whkg^−1^, a specific capacity of 1675 mAhg^−1^, a good rate performance, and a low cost of electrode materials [9,10]. In addition, the richness of sulfur as an active material and its environmentally friendly characteristics further enhance the economic and ecological attractiveness of LiSBs compared with the alkali metal-ion batteries [11]. As depicted in Figure 1, a typical lithium–sulfur battery configuration comprises a lithium metal anode, a sulfur-based cathode composite (consisting of an active material, conductive additives, and a polymeric binder), a microporous separator, and an electrolyte solution. The electrolyte is commonly formulated as an ether- or ester-based liquid, typically composed of a binary mixture of 1,3-dioxolane (DOL) and 1,2-dimethoxyethane (DME) [12].

However, the large-scale promotion and application of LiSBs still face some challenges [13,14], including the poor conductivity of the sulfur cathode [15] and the shuttle effect [16] of high-order lithium polysulfides (LiPSs, such as Li_2_S_8_, Li_2_S_6_, and Li_2_S_4_). The shuttle effect is primarily attributed to the high solubility of high-order LiPSs, which are generated during the charging and discharging cycles. These high-order LiPSs can eventually be transformed into insoluble low-order LiPSs (such as Li_2_S_2_ and Li_2_S) within the electrolyte. Furthermore, as the battery reaction proceeds, a barrier layer of Li_2_S will accumulate on the electrode [17]. This accumulation impedes the chemical reaction of the battery, leading to the progressive depletion of active substances and significantly reducing the battery life and coulomb efficiency.

Currently, designing anchoring materials on sulfur electrodes is considered to be a practical solution [18] to inhibit the shuttle effect, promote the round-trip circulation of active substances, and improve the life of LiSBs. A good anchoring material should possess the following characteristics [19,20]:

(1) It has excellent conductivity, facilitating the transmission of ions and electrons.
(2) It demonstrates a strong adsorption for LiPSs.
(3) It can lower the decomposition barrier of Li_2_S and facilitate the diffusion of Li ions on the surface.

Two-dimensional materials [21,22] have shown significant potential pertaining to anchoring materials, owing to their highly extended specific surface area and the high density of chemically active sites formed by the atomic-level exposure on the surface. At present, the doping of 2D materials with impurity atoms [23], thereby altering the electrical and chemical attributes of the materials, has also been shown theoretically and experimentally to improve the properties of 2D materials. As an illustration, Fei et al. investigated the impact of the doping of V_2_B_2_ with oxygen and discovered that the introduction of O atoms enabled the substrate to adsorb Li_2_S with a adsorption energy of up to 4.75 eV, significantly enhancing its catalytic activity for Li_2_S decomposition, thereby reducing the decomposition energy to 0.42 eV [24]. In addition, Xiao’s team studied Ti-doped 2D SnSe and discovered that the formation of strong Ti-S bonds enhances the interaction between the substrate and Li_2_S, increasing the adsorption energy to 4.01 eV and reducing the free energy of the sulfur reduction reaction (SRR) reaction by 3.27 eV [25].

Recently, a new 2D material called thgraphene has been reported [26]. Thgraphene exhibits excellent thermal stability, showing no significant deformation at a constant temperature of 350 K. It also demonstrates high electronic conductivity, with an electronic density of states near the Fermi energy level reaching approximately three states/eV. In addition, studies have shown that alkali metal ions exhibit low diffusion barriers on its surface, showing a low energy barrier of 0.06 eV for potassium. Furthermore, Wang and their colleagues confirmed that thgraphene shows good adsorption properties for sodium polysulfide when used as an anchor material in sodium–sulfur batteries [27]. However, research on the single-atom substitutional doping of thgraphene as a modified anchoring material for LiSBs has not been explored in previous investigations.

Based on the first-principles calculation approaches, B, N, P, and Al were initially selected as potential doping elements for thgraphene. By evaluating the cohesive energy and formation energy of the doped structure, suitable doping atoms were analyzed and selected. Subsequently, the adsorption behaviors of both pristine and doped systems toward Li_2_S were systematically investigated, and the underlying adsorption mechanisms were thoroughly explored through the charge density differences, charge transfer, and projected density of states (PDOS). Furthermore, the performance of the anchoring material in retaining Li_2_S adsorption under an external electric field was assessed. Finally, we discuss the diffusion mechanisms of Li ions and the energy barriers associated with Li_2_S decomposition on both pristine and doped substrates.

## 2. Computational Methods

The SIESTA (version 4.0) computational package [28] was used to carry out all first-principles simulations based on the density functional theory in this study. Within the generalized gradient approximation (GGA), the Perdew–Burke–Ernzerhof (PBE) functional [29] was used to model the exchange-correlation potential. Grimme’s DFT-D3 dispersion correction method [30] was also introduced to describe the van der Waals (vdw) interactions [31] with respect to Li_2_S clusters and the pristine/doped thgraphene substrates. To ensure accuracy, the expansion of the valence electron wave function utilizes a double-ξ basis group (DZP), and the cutoff energy was specified as 280 Ry [32]. To avoid systematic errors due to periodic mirror effects and to eliminate interactions between neighboring layers, a vacuum layer of 30 Å was set up along the z-direction. We created a 2×2×1 supercell, and a 5×5×1 K-point grid [33] was selected in the corresponding Brillouin zone during the calculation. A force convergence criterion of 0.02 eV/Å was applied during all structural relaxation procedures, and the electronic relaxation accuracy was set to 10^−5^ eV. For the reaction kinetic analyses, the Climbing Image Nudged Elastic Band (CI-NEB) method [34] was used to calculate the decomposition of Li_2_S and the diffusion energy barriers of Li ions on the pristine/doped thgraphene substrates.

The cohesive energy [35] of B/N/P/Al-doped thgraphene is defined as follows:

(1) $$E_{coh}=\frac{mE_{C}+nE_{X}-E_{dop-Th}}{m+n}$$

Here, m and n denote the number of C atoms and doped atoms, respectively, in doped thgraphene. $E_{C}$ signifies the energy associated with a carbon atom in isolation, while E_X_ (where X = B, N, P, or Al) denotes the energy of an isolated doped atom. Additionally, $E_{dop-Th}$ is the energy of the doped thgraphene. A positive cohesive energy suggests strong atomic bonding interactions with the doped thgraphene, which reflects the overall structural stability of the system.

The formation energy is commonly employed as a criterion to assess the experimental synthesizability of the materials and it is defined as [36]

(2) $$E_{f}=E_{dop-Th}-E_{Th}+\mu_{C}-\mu_{X}$$

where $E_{dop-Th}$ and $E_{Th}$ represent the energies of the doped and pristine thgraphene, respectively. $\mu_{C}$ is the average energy per C atom in optimized graphene, and $\mu_{X}$ (where X = B, N, P, or Al) represents the average energy of element X in common compounds. Here, $\mu_{B}$ denotes the mean energy per boron atom in borophene, $\mu_{N}$ is half the average energy of the nitrogen molecule, $\mu_{P}\;$ stands for the mean energy per phosphorus atom found in black phosphorene, and $\mu_{Al}$ is the mean energy per aluminum atom in the metallic aluminum crystal. The low formation energy indicates that the doping process is thermodynamically favorable and can occur spontaneously under the experimental environment.

The stability of the Li_2_S adsorption by the anchoring material can be reflected by the adsorption energy, determined by the equation as follows [37]:

(3) $$E_{ads}=E_{AM}+E_{Li_{2}S}-E_{AM+Li_{2}S}$$

where $E_{AM}$ represents the energy of the anchoring material, which could be a pristine or doped thgraphene substrate. While $E_{Li_{2}S}$ and $E_{AM+Li_{2}S}$ represent the energies of the isolated Li_2_S cluster and the adsorption system of the Li_2_S cluster adsorbed on the substrate, respectively.

The differential charge density is determined using the following expression [38]:

(4) $$\Delta \rho =\rho_{AM+Li_{2}S}-\rho_{AM}-\rho_{Li_{2}S}$$

where $\rho_{AM+Li_{2}S}$, $\rho_{AM}$, and $\rho_{Li_{2}S}$ represent the charge density of the Li_2_S adsorbed system, the anchoring material, and the isolated Li_2_S clusters. The corresponding differential charge density image is drawn by VESTA software (version 3.5) [39], and the Hirshfeld method was employed to quantify the transferred charge [40].

## 3. Results and Discussions

### 3.1. Structural and Electrical Properties of Pristine Thgraphene

**Figure 2:**
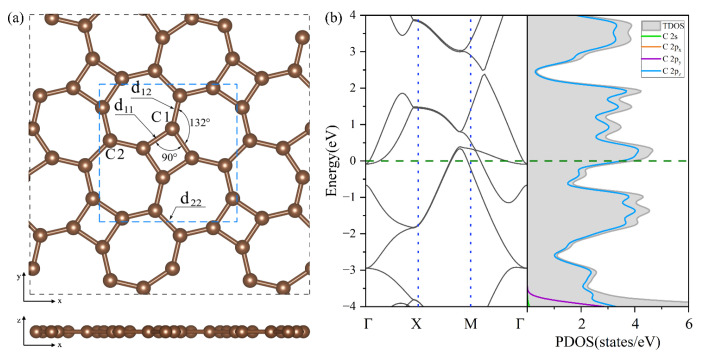
(**a**) The optimized geometry of thgraphene viewed from the top and side directions. (**b**) The band structure and PDOS of the unit cell of thgraphene, and the Fermi level is marked by the green dotted line.

In Figure 2a, thgraphene exhibits a single monolayer structure in which C atom rings are spread out alternately on the plane and belong to the P4/mbm space group, and its optimized lattice constant is a=b=5.79 Å. Thgraphene contains two types of carbon atoms in different chemical environments.

As noted in Figure 2a, one type is C1, which constitutes a four-membered carbon ring, and the other is C2, which only constitutes a seven-membered carbon ring. The bond lengths formed by the two kinds of carbon atoms are $d_{11}=1.45\;Å$, $d_{12}=1.41\;Å$, and $d_{22}=1.46\;Å$, while the angle of C1-C1-C1 is 90°, and the angle of C1-C1-C2 is 132°, supporting the conclusions of previous reports [26,27]. Figure 2b provides a view of the energy band structure and PDOS of the unit cell of thgraphene. A high density of electronic states is observed near the Fermi energy level, predominantly coming from the 2pz orbitals of carbon atoms. This indicates that thgraphene possesses a superior electrical conductivity similar to other anchoring materials, such as graphene [41], BP [42], and C_2_N [43]. Consequently, thgraphene contributes to a substantial rise in the electrical conductivity of the sulfur electrode, thus enhancing its charge transport properties.

### 3.2. Structure and Properties of Single-Atom Substitutionally Doped Thgraphene

To explore the effect of the impurity atom substitutional doping in thgraphene on its material properties, we selected elements located around carbon in the periodic table, such as B, N, P, and Al. These elements are abundant and readily obtainable in nature. Furthermore, the radii of their corresponding atoms do not differ significantly from that of the C atom, ensuring structural compatibility and facilitating the reduction in doping-induced lattice distortions. Additionally, the valence electron orbital energy levels of these neighboring elements are similar. This gradual adjustment of the electronic structure is more conducive to the synergistic optimization of the carrier concentration and mobility.

Considering the two distinct chemical environments of C atoms in the structure of thgraphene, the initial structures for the substitutional doping at the C1 and C2 positions with other elements are depicted in Figure S1. In this study, X_C1/C2_-thgraphene is designated to denote the doped substrate through the substitutional doping of thgraphene with a single atom X. To assess the stability of the doped system, the cohesive energy and formation energy were calculated based on Formulas (1) and (2). Summaries of the calculation outcomes can be found across Tables S1 and S2, providing insights into the energetic characteristics of the doped system. It is observed that structures with substitutional doping at the C1 site exhibit consistently higher cohesive energies than those at the C2 site. Specifically, substituting B, N, P, or Al at the C1 position results in cohesive energies of 9.69 eV, 9.72 eV, 9.61 eV, and 9.54 eV, respectively. Yang et al. investigated B and N doping in black phosphorus, obtaining cohesive energies of 7.57 eV and 9.22 eV [44], respectively. Meanwhile, Hernandez Cocoletzi et al. analyzed the effect of P and Al doping on silicene, obtaining a cohesive energy of 5.66 eV and 5.52 eV [45]. For comparison, their values are smaller than our results. This suggests that substitutional doping at the C1 site in the thgraphene substrate provides a superior thermodynamic stability compared to the C2 site.

As shown in Table S2, substitutional doping at the C1 site results in consistently lower formation energies compared to the C2 site. Specifically, B-, N-, P-, and Al-doped structures at the C1 position exhibit formation energies of −2.11 eV, −0.66 eV, 2.18 eV, and 8.78 eV, respectively. Li et al. reported that the formation energy of doping graphene with B atoms at different sites ranges from 3.61 eV to 7.15 eV [46], which is higher than that of B_C1_-thgraphene in this work. Kong et al. investigated N-doped borophosphene and reported a formation energy of −0.65 eV [42], which is also higher than N_C1_-thgraphene in our work. Dai et al. explored the substitutional doping of Al and P at different positions on penta-graphene, where the P or Al doping of carbon atoms in particular positions has lower formation energies [47]. These findings collectively suggest that the doping at the C1 site is more thermodynamically favorable and thus more likely to be achievable under experimental conditions. Therefore, only materials doped at the C1 position are selected for subsequent analyses in this study.

**Figure 3:**
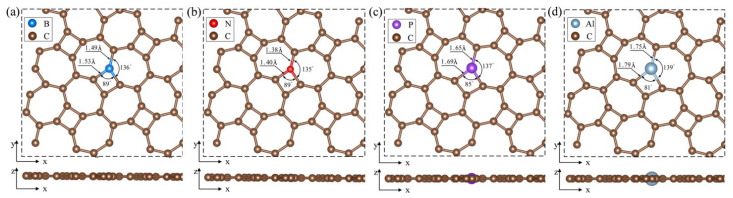
Optimized geometry of substitutional doping structure viewed from top and side directions. (**a**) BC1-thgraphene, (**b**) NC1-thgraphene, (**c**) PC1-thgraphene, and (**d**) AlC1-thgraphene.

Figure 3 displays the optimized configurations of B_C1_-thgraphene, N_C1_-thgraphene, P_C1_-thgraphene, and Al_C1_-thgraphene. In Figure 3a and Figure 3b, B_C1_-thgraphene exhibits tiny increases in the $d_{11}$ and $d_{12}$ bond length by 0.08 Å and 0.09 Å, respectively, compared to the pristine thgraphene. In contrast, the bond lengths of N_C1_-thgraphene remain almost unchanged. Given the similar size of C, B, and N atoms [48], the overall geometry of the B- and N-doped structures remain comparable to that of the pristine thgraphene. This structural similarity contributes to the enhanced stability and lower energies observed for B_C1_-thgraphene and N_C1_-thgraphene. As depicted in Figure 3c and Figure 3d, the larger radii of P and Al cause the $d_{11}$ bond lengths to increase to 1.69 Å and 1.79 Å, respectively. The corresponding $d_{12}$ bond lengths also increase to 1.65 Å and 1.75 Å, respectively. At the same time, the bond angles between P or Al and their neighboring C atoms decrease to 85° and 81°, respectively, which distorts the geometry of the thgraphene structure near the doping site. Al_C1_-thgraphene exhibits the highest formation energy, reaching up to 8.78 eV, indicating that it is difficult to synthesize experimentally. In summary, only B_C1_-thgraphene, N_C1_-thgraphene, and P_C1_-thgraphene are considered suitable doped anchoring materials for further investigation.

**Figure 4:**
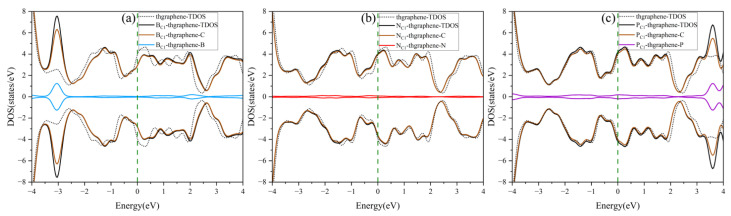
The density of states of the doped and pristine thgraphene. (**a**–**c**) correspond to the density of states for B_C1_-thgraphene, N_C1_-thgraphene, and P_C1_-thgraphene, respectively.

The DOS of the pristine and doping thgraphene is shown in Figure 4. As illustrated in Figure 4a, the doped B atom contributes significantly to the DOS primarily around an energy level of approximately −3 eV. At the same time, the TDOS of B_C1_-thgraphene exhibits a rightward shift compared to the that of the pristine system. As a charge acceptor atom, the B atom readily aids in the transfer of electrons from C atoms from lower to high energy levels. Consequently, some electronic states that were originally at a low energy become excited to higher energy states. This rearrangement of electronic energy typically results in a reduction in the overall stability of the whole system [49], as more energy is required to maintain the new distribution of electronic states. The differential charge density of the B_C1_-thgraphene system is shown in Figure S2a. The results indicate a localized region of charge accumulation (represented by yellow) around the B atom, which is surrounded by a region of charge depletion (shown in blue), suggesting that the B atom tends to draw electrons from its neighboring C atoms. Furthermore, the Hirshfeld charge analysis reveals that approximately 0.015|e| is transferred from the substrate to the doped atom, which likely enhances the interaction with the S atoms of the LiPSs.

The DOS of the N_C1_-thgraphene system is shown in Figure 4b, and the doped N contributes little to the total DOS of the system, and the shapes of the DOS curves remain basically the same before and after doping. Meanwhile, the doping-TDOS curve is shifted slightly to the left compared to that of the pristine thgraphene, implying that such doping renders the N_C1_-thgraphene structure energetically more favorable. Figure S2b demonstrates the differential charge density of N_C1_-thgraphene. The yellow regions representing the charge accumulation are located in the direction of the three C atoms bonded to the N atom. However, the bulk of the yellow regions is skewed towards the C atoms. The results of Hirshfeld charge calculations also show that a 0.028|e| charge was transferred from the doped atom to the substrate. The underlying cause of this phenomenon may appear to be that the outermost electron count of the substituted N atom is higher than that of the replaced C atom. The electron-rich nature of the doped N atom may lead to a repulsive interaction with S atoms in LiPSs.

The DOS of P_C1_-thgraphene is shown in Figure 4c. The doped P atom contributes to an increase in the density of electronic states at high energy levels around 3.5 eV, indicating that P doping introduces additional high-energy electrons to the substrate. The differential charge density of P_C1_-thgraphene is illustrated in Figure S2c, where a blue region representing charge depletion appears around the P atom, while yellow regions of charge accumulation are observed around the adjacent C atoms. Hirshfeld charge calculations reveal that 0.236|e| of charge is transferred from the P atom to the substrate. Due to this charge transfer, the doped P site can act as a positive electric center, and the resulting local charge polarization may enhance the adsorption interaction toward LiPSs.

### 3.3. Optimized Structures of Li_2_S Adsorption on Pristine and Doped Thgraphene

Figure S3a presents the optimized structure of the free Li_2_S cluster, which adopts a planar chain configuration. Two Li atoms are symmetrically arranged around the S atom, with a Li-S bond of 2.12 Å and a Li-S-Li bond angle of 118.4°, which is consistent with previous reports [20]. Figure S3b,c show the optimized molecular structures of the primary electrolyte components, 1,3-dioxolane (DOL) and 1,2-dimethoxyethane (DME) [50], in LiSBs. Figure S3d,e show the optimized adsorption structures of Li_2_S with DOL and DME, indicating that Li atoms primarily interact with O atoms in DOL and DME. Based on Formula (3), for Li_2_S interacting with DOL and DME, the adsorption energies were measured at 1.67 eV and 1.75 eV, respectively. Therefore, an effective anchoring material should exhibit an adsorption energy higher than 1.75 eV to achieve robust immobilization and prevent the migration and diffusion of Li_2_S in the electrolyte.

**Figure 5:**
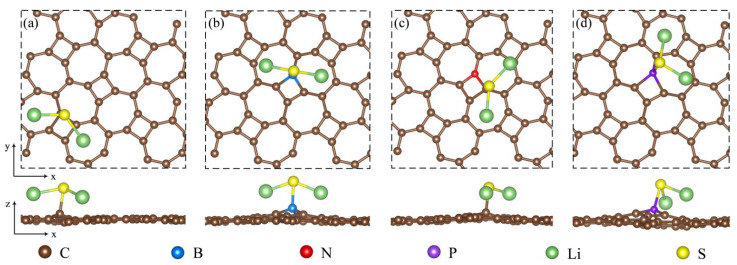
An illustration of the top and side views showing the most stable adsorption configuration for Li_2_S adsorbed on (**a**) pristine thgraphene, (**b**) B_C1_-thgraphene, (**c**) N_C1_-thgraphene, and (**d**) P_C1_-thgraphene.

To ascertain the optimal adsorption conformations, Li_2_S was positioned at various adsorption sites on pristine and doped thgraphene (B-, N-, and P-doped) with different initial orientations, and the relevant adsorption energies were derived. Representative optimized structures are presented in Figures S4–S7. Based on the magnitude of the adsorption energy, the most stable adsorption configurations on each substrate were selected in Figure 5 for an in-depth investigation.

As shown in Figure 5, Li_2_S is adsorbed onto these substrates while preserving the structural characteristics of the original clusters. The Li_2_S clusters tend to adsorb onto the substrate surface in a parallel orientation. In Figure 5a,c, the S atom preferentially occupies the top sites above C atoms, while the Li atoms are more inclined to adsorb at the hollow positions located in the heart of the seven-membered ring. The S atom does not form a bond with the N atom. This is likely because the differential charge density results indicate that the N-doped site is a region of charge accumulation, forming a negative charge center. Since the outer shell of the S atom is electron-rich, a repulsive interaction exists between the S atom and this negatively charged region. As indicated in Table 1, this is the underlying cause. The adsorption energy of Li_2_S on N_C1_-thgraphene (3.13 eV) is close to that on the pristine thgraphene (3.10 eV), and the difference in the adsorption distance between the two substrates is only 0.02 Å. While in Figure 5b,d, it demonstrates that Li_2_S is mainly adsorbed by B and P on the doped substrates, with the S atom located exactly at the top position of the B or P atom. According to Table 1, the adsorption energies of Li_2_S on B_C1_-thgraphene and P_C1_-thgraphene are 3.53 eV and 4.48 eV, respectively, exceeding the value of the pristine thgraphene. This can be attributed to the formation of a more favorable polarized field at the doping sites after the B and P doping, which facilitates an attractive interaction with the S atom. This has also been confirmed by the earlier differential charge density results for doping substrates.

The adsorption energy of Li_2_S on both pristine and doped substrates is higher than that on electrolyte solution molecules, which contributes to the inhibition of the shuttle effect. Moreover, the adsorption energies are also greater than those reported for WS_2_ (1.36 eV) [51], biphenylene (2.55 eV) [37], and BC_2_N (1.60 eV) [52] substrates. This reflects the excellent anchoring ability of both pristine and doped thgraphene, as well as the stability of the interface formed in the adsorption system. Notably, B and P doping significantly enhance this performance.

### 3.4. Charge Transfer Between Li_2_S and Substrate Materials

**Figure 6:**
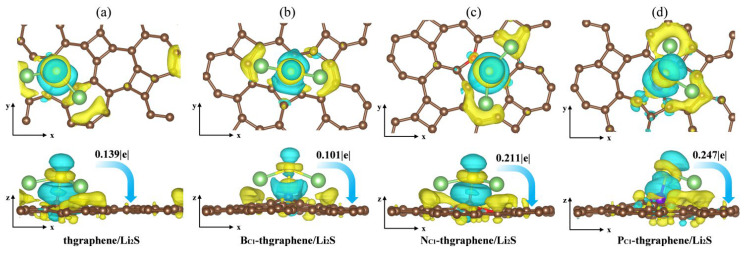
The differential charge density of the adsorption system (**a**) thgraphene; (**b**) B_C1_-thgraphene; (**c**) N_C1_-thgraphene; (**d**) P_C1_-thgraphene, and the isosurface level is set to be 0.0035 e${Å}^{-3}$. The light blue area indicates electron depletion, whereas the yellow area signifies electron accumulation.

Aiming to reveal the underlying adsorption behaviors of Li_2_S on the substrates, we calculated the charge transfer and plotted the differential charge density distribution. As presented in Figure 6a, there is a notable yellow charge gain region around the S atom, and the Hirshfeld charge transfer calculations indicate that the Li_2_S cluster transfers 0.139|e| of charge to thgraphene substrates. Meanwhile, a yellow charge gain region appears on the substrate surface directly below the Li atom, and the calculation reveals that the Li atom transfers a charge to the substrate and the S atom at the same time, which further loses electrons to transition to Li^+^, suggesting that Li_2_S shows an obvious charge polarization effect [53]. It weakened the Li-S bond to a certain extent.

As shown in Figure 6b, although the charge transfer between Li_2_S and B_C1_-thgraphene decreases to 0.101|e|, more charge-gaining regions appear near the B atom and extend toward the space between the B and S atoms, while charge-depletion regions are located near the S atom and adjacent to the charge-gaining areas. Furthermore, the B-S bond length reaches 2.02 Ǻ, likely due to the tendency of the B atom’s 2p lone-pair orbital to hybridize with the 3p lone pair of electrons of the S atom [54], forming a strong B-S bond that exhibits characteristics intermediate between ionic and covalent bonding. The localized positive charge introduced by the B atom, along with the formation of this B-S bond, enhances the anchoring ability of B_C1_-thgraphene compared to pristine thgraphene.

As shown in Figure 5c,d, more charge transfer occurs in both N_C1_-thgraphene/Li_2_S and P_C1_-thgraphene/Li_2_S systems. The amounts of charge transferred from the Li_2_S cluster to substrates are 0.211|e| and 0.247|e|, respectively. In the case of N_C1_-thgraphene, an electron-rich region is formed that tends to donate electrons to the S atom [55]. This leads to repulsion with the inherently electron-rich nature of the S atom itself. Despite the charge transfer, its direction contradicts the electrostatic complementary expected for strong adsorption, thereby weakening the N-S interaction. As seen in Figure 5d, the larger atomic radius of P increases the bond lengths between P and its neighboring C atoms, resulting in a more dispersed electron cloud. This facilitates the formation of stable bonds with the lone-pair electrons of S through lone pair–lone pair interactions [47]. Furthermore, the calculated differential charge density reveals that P-doped thgraphene generates a charge-depletion region, promoting the formation of a robust adsorption system for Li_2_S. Consequently, P_C1_-thgraphene demonstrates an adsorption energy of up to 4.48 eV, surpassing the values observed for the other three substrates.

### 3.5. The Electronic DOS of the Adsorption System

**Figure 7:**
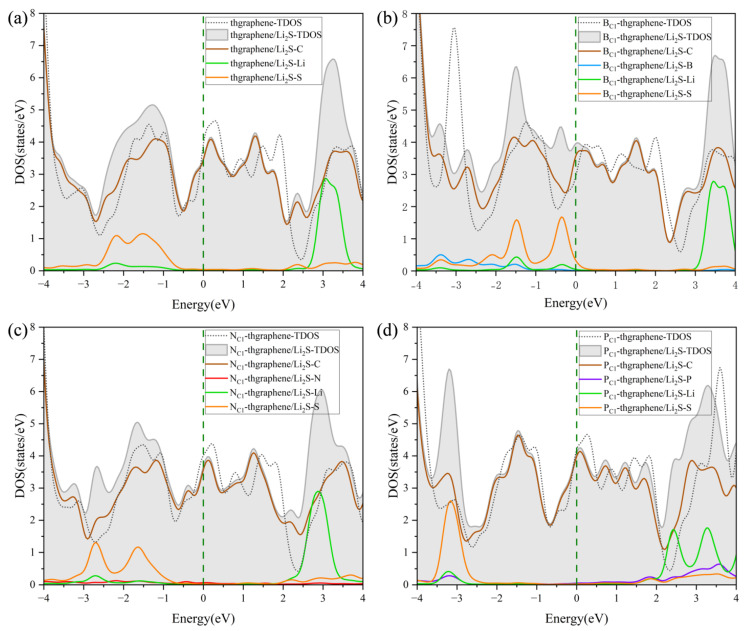
The DOS of Li_2_S adsorbed on (**a**) pristine thgraphene, (**b**) B_C1_-thgraphene, (**c**) N_C1_-thgraphene, and (**d**) P_C1_-thgraphene; the Fermi level is marked by the dark green dashed line.

To investigate how the adsorption of Li_2_S on pristine thgraphene, B_C1_-thgraphene, N_C1_-thgraphene, and P_C1_-thgraphene influences the electronic conductivity of the system, the DOS was calculated for all four configurations, as presented in Figure 7.

It can be observed that all four adsorption configurations maintain good metallic characteristics. Each configuration exhibits a good electronic DOS at the Fermi level, which is highly beneficial for maintaining the good electrode conductivity during the operating of LiSBs. The DOS curves after adsorption show slight shifts in the peaks to the left compared to those before adsorption, which further confirms the structural stability of Li_2_S after adsorption [49]. The DOS contributed by Li is mainly distributed in the energy range of 2~4 eV, while that contributed by S is mainly located in the negative energy range.

As shown in Figure 7a, the overlap between the PDOS of Li and C at higher energy levels indicates the formation of an orbital hybridization between them, which facilitates the adsorption of Li onto the substrate. Meanwhile, the PDOS contribution from the S atom appears at the deeper part of the valence band, implying that the S atom also interacts with the substrate during the adsorption of Li_2_S. In Figure 7b,d, it is evident that the PDOS of B and P atoms overlaps with the DOS curves of Li or S atoms, suggesting that B and P form certain chemical interactions with Li or S during the adsorption process. This indicates that B_C1_-thgraphene and P_C1_-thgraphene exhibit a strong adsorption toward Li_2_S, demonstrating that B and P doping significantly improves the anchoring ability of the material. The DOS in Figure 7c is similar to that of Figure 7a, confirming that N does not play a major role in the adsorption process of Li_2_S. This observation is consistent with the optimized adsorption structure shown in Figure 5c.

### 3.6. Modulation Adsorption Mechanisms by External Electric Field

**Figure 8:**
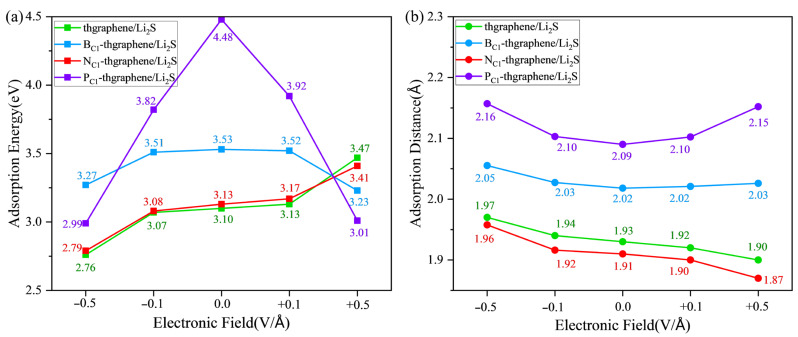
The adsorption energies (**a**) and adsorption distances (**b**) of Li_2_S on four kinds of substrates under the external electric field.

When the LiSBs are operating under normal conditions, an electric field is generated in the direction perpendicular to the anchoring material, and this field varies with the battery reaction [56]. It is therefore imperative to examine the anchoring capability and structural stability of the Li_2_S adsorption system under the external electric field. Based on previously reported studies involving applied electric fields [57], this work sets the electric field strength to −0.5 V/Å, −0.1 V/Å, +0.1 V/Å, and +0.5 V/Å, with the direction being perpendicular to the surface of the 2D material. The vector pointing from −z to +z is defined as the positive direction of the electric field. Figure 8 illustrates the adsorption energies and adsorption distances of Li_2_S on pristine thgraphene, B_C1_-thgraphene, N_C1_-thgraphene, and P_C1_-thgraphene substrates under the electric field. The corresponding differential charge density under the electric field is shown in Figure S8, Figure S9, Figure S10, and Figure S11, respectively.

By synthesizing the results from Figure 8, Figures S8 and S10, it is evident that the impacts of the external electric field on the adsorption of Li_2_S on pristine and N-doped thgraphene are similar. In the absence of an applied electric field, a negative electric field causes a reduction in the adsorption energy, an increase in the adsorption distance, and a decrease in the amount of electron transfer to the substrate. Conversely, a positive electric field increases the adsorption energy, shortens the adsorption distance, and increases the amount of charge transferred to the substrate. For example, as indicated by the green line in Figure 8 and Figure S8, under the application of a −0.5 V/Å negative electric field, the adsorption energy decreases to 2.76 eV, which is 0.34 eV lower than that without an electric field. Meanwhile, the adsorption distance increases to 1.97 Å (an increase of 0.04 Å), and the transferred charge amount decreases to 0.039|e| (a reduction of 0.10|e|). In the presence of a 0.5 V/Å positive electric field, the adsorption energy reaches 3.47 eV, which is 0.37 eV higher than that without an electric field. The adsorption distance decreases to 1.90 Å (a reduction of 0.03 Å), and the transferred charge amount increases to 0.332|e| (an increase of 0.193|e|). Previous findings have already demonstrated that the adsorption effects of Li_2_S on pristine and N-doped thgraphene are highly similar. The resulting electric polarization effect under the external electric field is also consistent.

Considering the results presented in Figure 8, as well as Figures S9 and S11 comprehensively, the impact of an external electric field on the adsorption of Li_2_S on B-doped and P-doped thgraphene exhibits similarities. Relative to the no-field condition, a negative electric field lowers the adsorption energy, increases the adsorption distance, and decreases the amount of transferred charge to the substrate. Conversely, a positive electric field also reduces the adsorption energy and increases the adsorption distance but increases the amount of transferred charge to the substrate. For instance, as indicated by the purple line in Figure 8 and Figure S11, when a negative electric field of −0.5 V/Å is applied, the adsorption energy decreases to 2.99 eV, which is 1.49 eV lower than that in the no-field condition. The adsorption distance increases to 2.16 Å, an increment of 0.07 Å, and the transferred charge decreases to 0.101|e|, a reduction of 0.146|e|. Upon applying a 0.5 V/Å positive electric field, the adsorption energy reaches 3.01 eV, which is a decrease of 1.47 eV compared to that in the no-field condition. The adsorption distance decreases to 2.15 Å, a reduction of 0.06 Å, and the transferred charge increases to 0.395|e|, an increment of 0.148|e|. Previous research findings have already demonstrated that the adsorption effects of B-doped and P-doped thgraphene on Li_2_S are primarily influenced by the doped atoms. The impact of the electric field here is also consistent. It is important to note that for both substrates, in the presence of a +0.5 V/Å electric field, although the adsorption energy between Li_2_S and the substrate decreases, the amount of transferred charge to the substrate actually increases. This should be attributed to the polarization effect of the electric field, where a positive electric field is more conducive to the charge transfer to the substrate, while a negative electric field hinders it. Even the results in Figure S9 show that at −0.5 V/Å, 0.064|e| of charge is transferred from the substrate to Li_2_S. Similar results induced by an externally applied electric field have also been reported in the other published literature [57,58].

An analysis of all results under the external electric field shows that the adsorption of Li_2_S on the above four substrates continues to outweigh its interaction with the electrolytes (DOL and DME), demonstrating a robust anchoring capability. Furthermore, the DOS image in Figure S12 shows that adsorption systems under the external electric field still have a high density of electronic states close to the Fermi level, suggesting that four substrates always maintain excellent conductivity.

### 3.7. Diffusion Properties of Li on the Pristine and Doped Thgraphene Substrate

**Figure 9:**
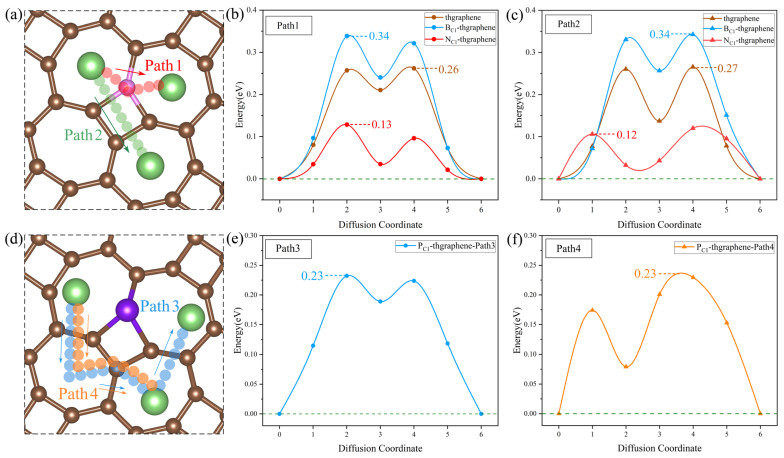
(**a**) The possible diffusion paths of Li on the pristine, B-, and N-doped thgraphene. The pink atoms represent C/B/N. (**b**) Li diffusion energy barriers along Path1 on the pristine, B-, and N-doped thgraphene. (**c**) Li diffusion energy barriers along Path2 on the pristine, B-, and N-doped thgraphene. (**d**) The possible diffusion paths of Li on P_C1_-thgraphene. (**e**) Li diffusion energy barriers along Path3 on the P_C1_-thgraphene substrate. (**f**) Li diffusion energy barriers along Path4 on the P_C1_-thgraphene substrate.

The diffusion energy barrier of Li ions on the substrate determines their migration rate and influences the capacity retention at a high current density. Meanwhile, the rapid diffusion of Li can enhance the conversion reaction kinetics of LiPSs, suppress their dissolution and shuttling, and thereby improve the sulfur utilization and electrochemical stability upon repeated cycling [59]. In this work, the diffusion energy barrier was calculated using the CI-NEB method. The calculations are based on the diffusion of a Li ion from a stable adsorption site to another near equivalent site. Considering the structural characteristics of the thgraphene and the influence of doping, the diffusion paths for the four types of substrates are set as shown in Figure 9a and Figure 9d, respectively. In Figure 9a,d, the diffusion paths depicted by the four different-colored small spheres represent the optimal diffusion paths obtained through calculations. Figure 9a illustrates the actual diffusion paths (Path1 and Path2) corresponding to the pristine substrate, as well as the B- and N-doped substrates. In contrast, Figure 9d shows the actual diffusion paths (Path3 and Path4) corresponding to the P-doped substrate.

As shown in Figure 9a, the migration path for Path1 is indicated by red small spheres. In this pathway, the Li ion directly migrates to the center of the adjacent seven-membered ring through a C, B, or N atom. For Path2, the migration path is marked by green small spheres, where Li passes through the four-membered ring containing a C, B, or N atom at the C1 position before migrating to the center of the seven-membered ring. According to the calculations, as shown in Figure 9b,c, the diffusion barriers for both paths are nearly identical across the three substrates. Specifically, on the pristine substrate, the diffusion barriers for Path1 and Path2 are approximately 0.26 eV. On the B-doped substrate, both paths exhibit a barrier of around 0.34 eV. It is mainly due to the formation of a small positive region near the B atom (shown in Figure S2a), which causes Li to be repelled by electrostatic forces and makes it difficult to migrate across the B atom. In contrast, on the N-doped substrate, a small negative region formed by the N atom (shown in Figure S2b) can attract Li ions, and the diffusion barriers for both paths are significantly lower, at 0.12 eV.

Despite having substrates of the same structure and setting the same initial diffusion model, after the P doping, the actual diffusion path of Li on the substrate differs from that of the previous three substrates. As shown in Figure 9d, the actual diffusion Path3 exhibits a “W”-shaped pattern. Starting from the most stable initial position, it passes through a seven-membered ring twice, bypasses the four-membered ring where P is located, and then reaches the end position. The diffusion barrier is shown to be 0.23 eV in Figure 9e. Similarly, the actual diffusion Path4 also bypasses the four-membered ring where P is located and then reaches the end position, with the corresponding diffusion barrier of 0.23 eV displayed in Figure 9f. Such diffusion paths of Li on the P-doped substrate should be attributed to the relatively significant change in the local positive potential field around the four-membered ring induced by the P doping. This strong positive potential impedes the Li migration towards the P atom, as evidenced by the results in Figure S2c.

The diffusion energy barriers of Li on all four substrates are lower than 0.35 eV, which are lower than those on the materials of graphene (0.37 eV) [60] and GeP_3_ (0.50 eV) [61]. This indicates that both the pristine and doped thgraphene exhibit a better enhancement of Li diffusion kinetics.

### 3.8. Decomposition of Li_2_S on Pristine and Doped Thgraphene Substrates

**Figure 10:**
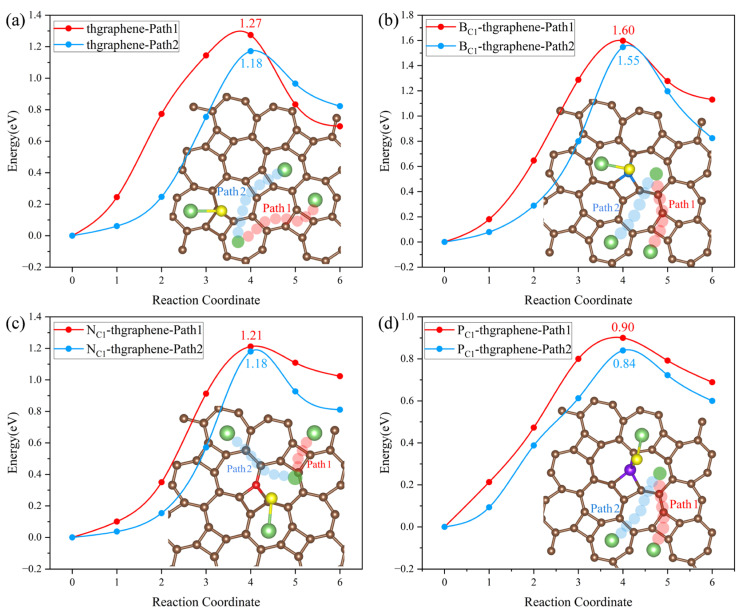
Decomposition paths of Li_2_S and decomposition energy barriers on the (**a**) pristine thgraphene, (**b**) B_C1_-thgraphene, (**c**) N_C1_-thgraphene, and (**d**) P_C1_-thgraphene substrates.

During the operation of LiSBs, the decomposition kinetics of Li_2_S, the end product of polysulfides, is an important factor affecting the battery multiplication performance. Li_2_S adsorbed on the anchoring material must release active substances such as Li^+^ through the breaking of Li-S bonds as soon as possible to prevent the excessive accumulation of Li_2_S from degrading the reversible capacity [20]. In this work, to determine the decomposition energy of Li_2_S on the substrates, we established the initial model based on the stable adsorption configurations of Li_2_S on the four types of substrates, as illustrated in Figure 5. Subsequently, we considered the scenario where one of the Li-S bonds breaks, and the Li atom diffuses to the nearest most stable adsorption site nearby. As shown in Figure 10, two possible decomposition paths are selected on four substrates. The energy required for this process is defined as the decomposition energy barrier, obtained through CI-NEB calculations.

In Figure 10a, Path2 is identified as the optimal decomposition path for Li_2_S on the pristine thgraphene surface, with an energy barrier of 1.18 eV. Figure 10b–d illustrate that Li_2_S also tends to choose Path2 as the optimal decomposition path, which does not cross the four-membered ring at the B/N/P-doped position. The decomposition energy barrier on the B_C1_-thgraphene substrate is 1.55 eV, surpassing that of the pristine thgraphene by 0.37 eV. The optimized decomposition energy barrier on the N_C1_-thgraphene substrate is the same as that on the pristine thgraphene. The doping of the P atom reduces the decomposition energy, which is only 0.84 eV lower than those of graphene (1.81 eV) [62], Ti_2_CS_2_ (1.51 eV) [63], and BIP (1.43 eV) [37]. As discussed above, the electrostatic repulsion between P and Li drives the efficient migration of Li. In addition, the Li-S bond length (2.46 Å) in P_C1_-thgraphene/Li_2_S is longer than that in the thgraphene/Li_2_S (2.37 Å) adsorption system. The weakened Li-S bond is easier to break.

### 3.9. Lithium Storage on Pristine and Doped Thgraphene Surface

Additionally, if the anchoring material has a good lithium storage capacity, the Li adsorbed on the substrate surface during the battery cycle will increase contact with higher-order polysulphide clusters, promoting their conversion to lower-order polysulphides, which are ultimately reduced to Li_2_S. This process can effectively mitigate the shuttle effect of polysulphide clusters, promote the recycling and regeneration of active materials, and thereby enhance the reaction kinetics of LiSBs.

**Figure 11:**
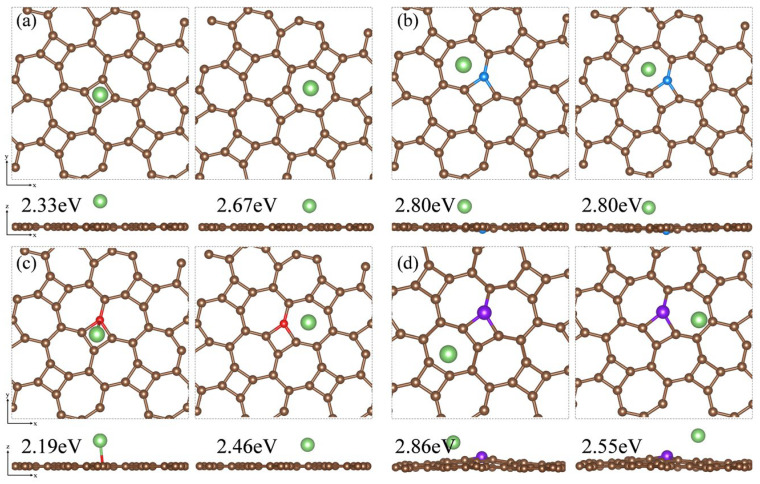
Top and side views of the Li atom adsorbed on the surface of (**a**) pristine thgraphene, (**b**) B_C1_-thgraphene, (**c**) N_C1_-thgraphene, and (**d**) P_C1_-thgraphene and their adsorption energies.

Based on the adsorption configuration of Li_2_S, we initially placed the Li atom at two relatively stable adsorption sites: the center of the four-membered carbon ring and the center of the seven-membered carbon ring on the substrate surface. DFT optimization results and adsorption energy calculations indicate that Li atoms tend to be adsorbed at the center of the seven-membered carbon ring. In Figure 11a, the adsorption energy of the thgraphene storing one Li atom is 2.67 eV, which is higher than that of BSi_4_ (1.50 eV) [64] and Cu_2_Si (2.40 eV) [65], demonstrating that thgraphene possesses a superior adsorption strength for Li. In Figure 11b,d, the local positive charge centers introduced by B and P atoms exert a noticeable repulsive effect on Li, causing the Li atom to preferentially adsorb at stable sites distant from B and P. The adsorption energies of the Li atom on B and C are as high as 2.80 eV and 2.86 eV, respectively, indicating that doping with B and P atoms enhance the lithium storage capacity of the substrate. In Figure 11c, the adsorption energy of Li on the N surface is 2.46 eV, which is slightly lower than that of the pristine thgraphene.

## 4. Conclusions

Both pristine and B-, N-, and P-doped thgraphene have been investigated. Our results illustrate that they all exhibit excellent structural stability and electrical conductivity, even when modulated by an external electric field. Furthermore, four substrates exhibit both a strong Li_2_S anchoring capability and enhanced catalytic activity towards Li_2_S decomposition when employed as anchor materials in LiSBs. Additionally, B, N, and P, as different dopants, have distinct effects on the electrochemical properties of thgraphene: (1) B introduces a local positive electric center at the doping site and promotes the charge gain around it. The B-S bonding, acting as a hybrid bonding intermediate between ionic and covalent bonding, greatly enhances the anchoring ability to Li_2_S. However, B-doping appropriately increases the diffusion difficulty of Li. (2) With its unique valence electron distribution and similarity to the C atom, N forms a strong, stable, and low-energy doped structure. Due to the presence of lone electron pairs in N, the N_C1_-thgraphene does not significantly improve the anchoring ability but slightly aids in reducing the diffusion barrier of Li on the substrate. (3) P is considered to be an excellent dopant. Through the rich charge transfer between P and the substrate, P forms an obvious positive center, and the stable bond formed by P-S shows great adsorption properties. In addition, P_C1_-thgraphene has a lower diffusion energy barrier for Li and a lower decomposition energy barrier for Li_2_S, which may make P_C1_-thgraphene the most ideal anchoring material among B-, N-, and P-doped thgraphene substrates.

## Figures and Tables

**Table 1:** Adsorption energy and adsorption distance of Li_2_S on pristine/doped thgraphene.

	Thgraphene	B_C1_-Thgraphene	N_C1_-Thgraphene	P_C1_-Thgraphene
Adsorption energy (eV)	3.10	3.53	3.13	4.48
Adsorption distance (Å)	1.93	2.02	1.91	2.09

## Data Availability

The original contributions presented in this study are included in the article/Supplementary Materials. Further inquiries can be directed to the corresponding author.

## Supplementary Materials

The following supporting information can be downloaded at https://www.mdpi.com/article/10.3390/ma18143269/s1: Figure S1: Initial structures of substitutional doping at C1 (a) and at C2 positions (b). Brown atom represents C atom and pink atom represents doped atom; Figure S2: Differential charge density diagram of single atom substitutional doped thgraphene, and yellow represents electron accumulation, while light blue represents electron depletion. (a) B_C1_-thgraphene, (b) N_C1_-thgraphene, and (c) P_C1_-thgraphene; Figure S3: (a) Optimized structure of Li_2_S. (b) Optimized molecules of DOL. (c) Optimized molecules of DME. (d) Li_2_S is adsorbed by DOL. (e) Li_2_S is absorbed by DME; Figure S4: Top and side views of the adsorption configurations and adsorption energies of Li_2_S adsorbed on the intrinsic thgraphene; Figure S5: Top and side views of the adsorption configurations and adsorption energies of Li_2_S adsorbed on B_C1_-thgraphene; Figure S6: Top and side views of the adsorption configurations and adsorption energies of Li_2_S adsorbed on N_C1_-thgraphene; Figure S7: Top and side views of the adsorption configurations and adsorption energies of Li_2_S adsorbed on P_C1_-thgraphene; Figure S8: Differential charge density of pristine thgraphene/Li_2_S under the external electronic field and the isosurface level is set to be 0.0035 e${Å}^{-3}$. The light blue region represents electron depletion and the yellow region represents electron accumulation; Figure S9: Differential charge density of B_C1_-thgraphene/Li_2_S under the external electronic field and the isosurface level is set to be 0.0035 e${Å}^{-3}$. The light blue region represents electron depletion and the yellow region represents electron accumulation; Figure S10: Differential charge density of N_C1_-thgraphene/Li_2_S under the external electronic field and the isosurface level is set to be 0.0035 e${Å}^{-3}$. The light blue region represents electron depletion and the yellow region represents electron accumulation; Figure S11: Differential charge density of P_C1_-thgraphene/Li_2_S under the external electronic field and the isosurface level is set to be 0.0035 e${Å}^{-3}$. The light blue region represents electron depletion and the yellow region represents electron accumulation; Figure S12: Density of states of Li2S absorbed on (a) thgraphene, (b) BC1-thgraphene, (c) NC1-thgraphene, (d) PC1-thgraphene under the external electric field. The Fermi level is marked by the dark green dashed line; Table S1: Cohesive energy of systems with different doping positions and Table S2: Formation energy of systems with different doping positions.
