# Supplementary material for: Adsorption and Decomposition Mechanisms of Li2S on 2D Thgraphene Modulated by Doping and External Electrical Field
Source: Materials (Basel). 2025 Jul 10;18(14):3269. doi: 10.3390/ma18143269 (PMC12300267; doi:10.3390/ma18143269)
Supplement: Supplementary file 1 [file materials-18-03269-s001.zip › materials-3697439-supplementary.pdf]

Supplementary Materials

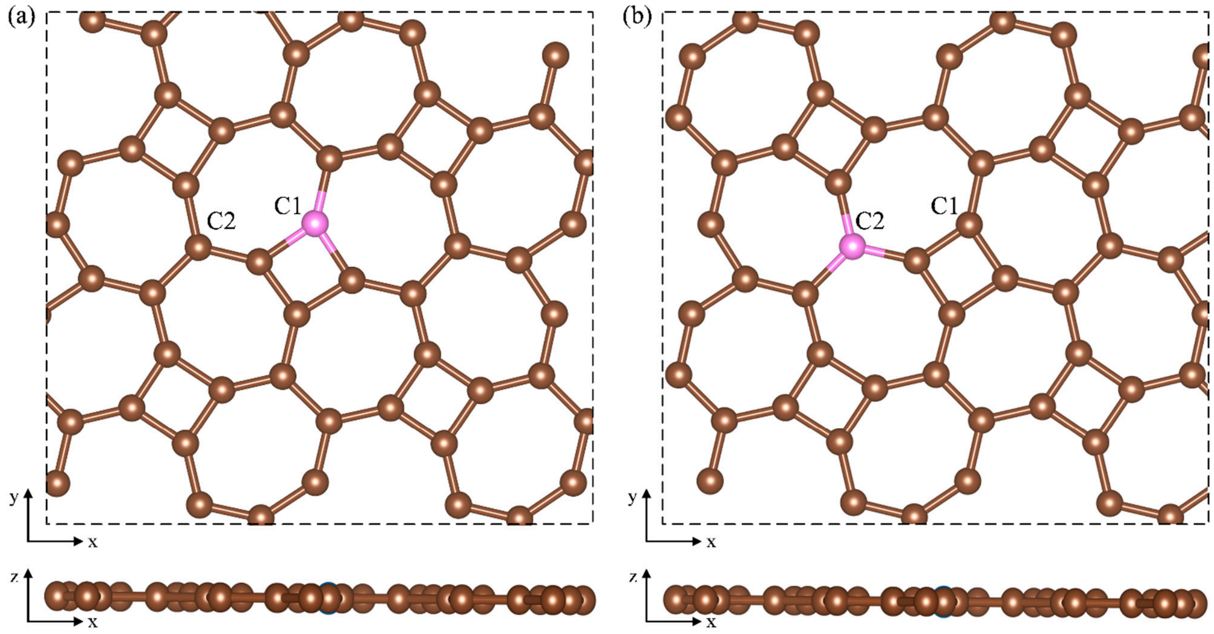

Figure S1. Initial structures of substitutional doping at C1 **(a)** and at C2 positions **(b)**. Brown atom represents C atom and pink atom represents doped atom.

Table S1. Cohesive energy of systems with different doping positions.

	$B_{C1/C2}$-thgraphene	$N_{C1/C2}$-thgraphene	$P_{C1/C2}$-thgraphene	$Al_{C1/C2}$-thgraphene
C1 position	9.69eV	9.72eV	9.61eV	9.54eV
C2 position	9.68eV	9.71eV	9.60eV	9.52eV

Table S2. Formation energy of systems with different doping positions.

	$B_{C1/C2}$-thgraphene	$N_{C1/C2}$-thgraphene	$P_{C1/C2}$-thgraphene	$Al_{C1/C2}$-thgraphene
C1 position	-2.11eV	-0.66eV	2.18eV	8.78eV
C2 position	-1.73eV	-0.24eV	2.34eV	9.47eV

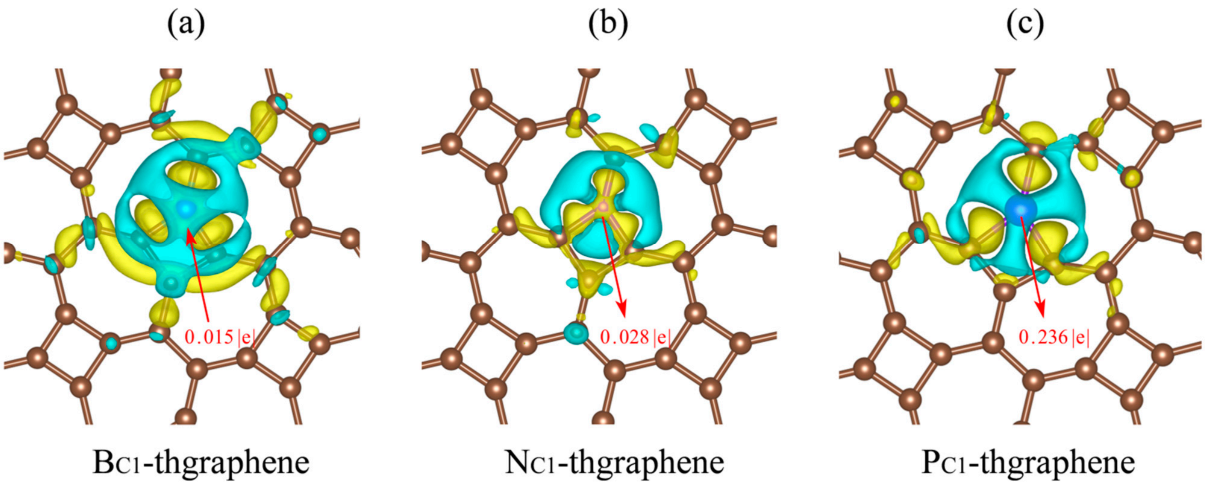

Figure S2. Differential charge density diagram of single atom substitutional doped thgraphene, and yellow represents electron accumulation, while light blue represents electron depletion. **(a)**B_{C1}-thgraphene, **(b)**N_{C1}-thgraphene, and **(c)**P_{C1}-thgraphene.

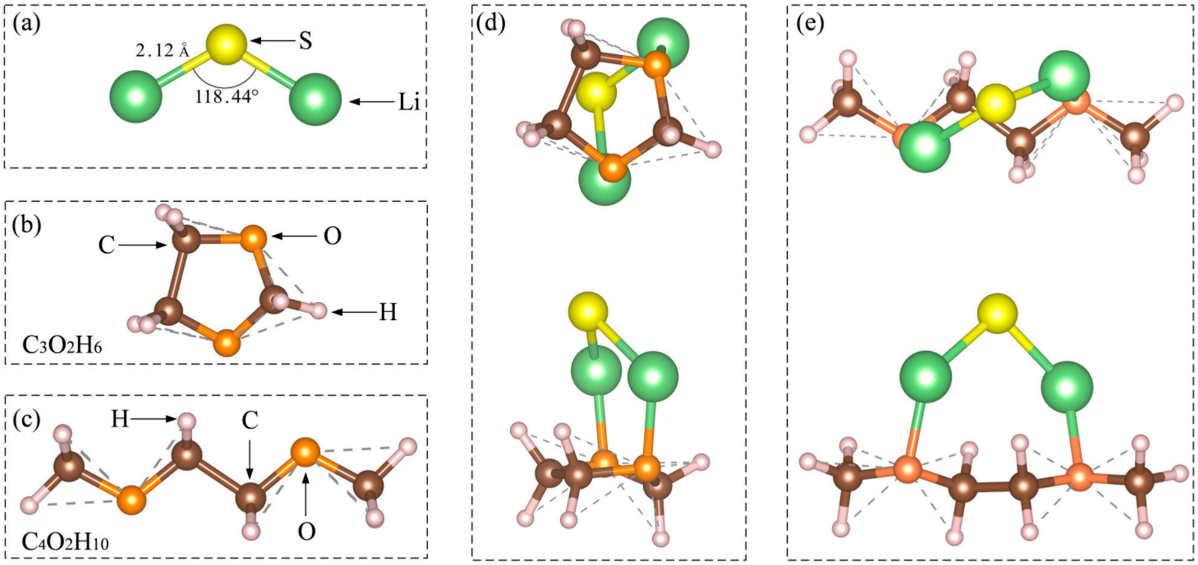

Figure S3. (a) Optimized structure of Li_2S. **(b)**Optimized molecules of DOL. **(c)**Optimized molecules of DME. **(d)**Li_2S is adsorbed by DOL. **(e)**Li_2S is absorbed by DME.

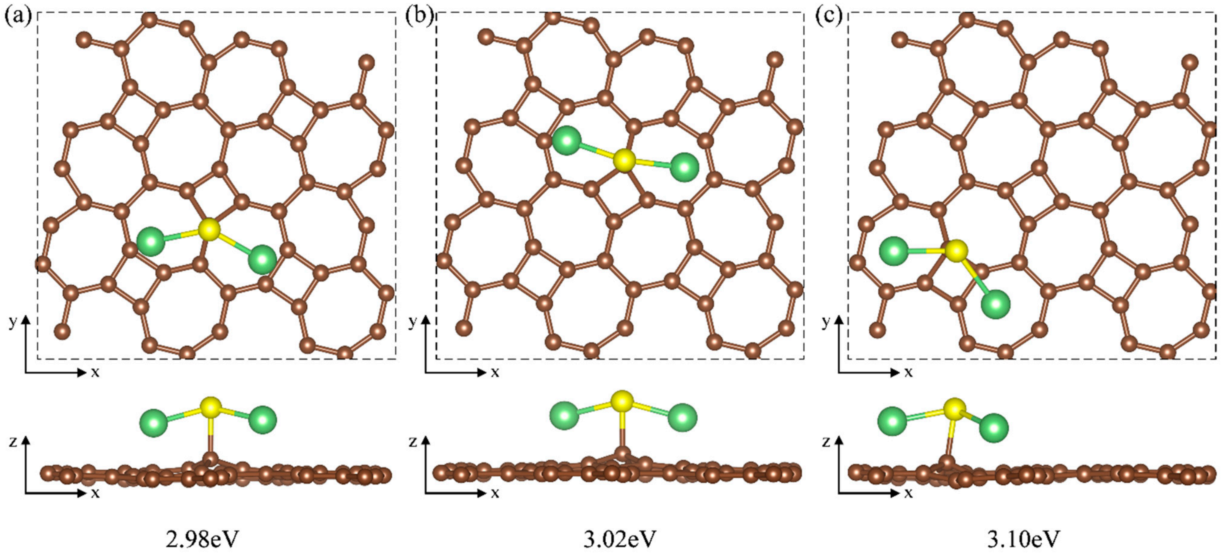

Figure S4. Top and side views of the adsorption configurations and adsorption energies of Li_2S adsorbed on the intrinsic thgraphene (**a**–**c**).

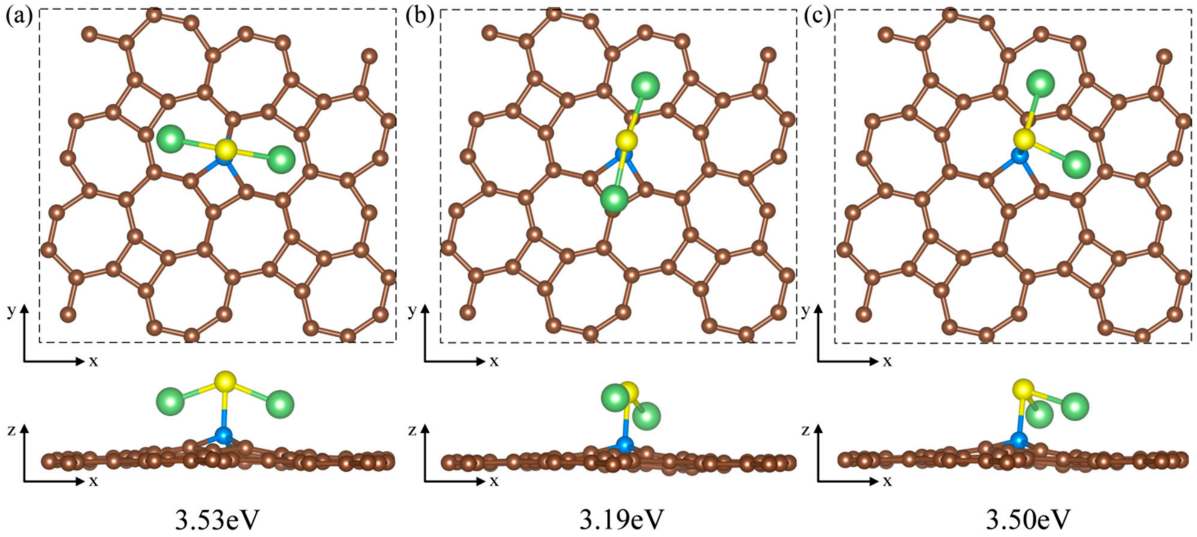

Figure S5. Top and side views of the adsorption configurations and adsorption energies of Li_2S adsorbed on B_{C1}-thgraphene (**a**–**c**).

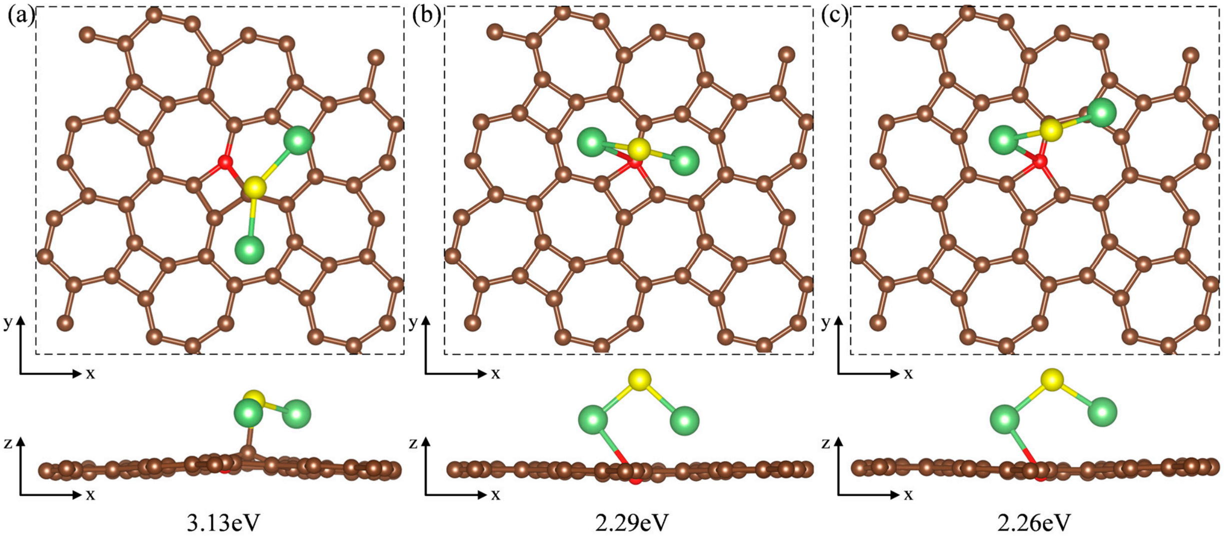

Figure S6. Top and side views of the adsorption configurations and adsorption energies of Li_2S adsorbed on N_{C1}-thgraphene (**a**–**c**).

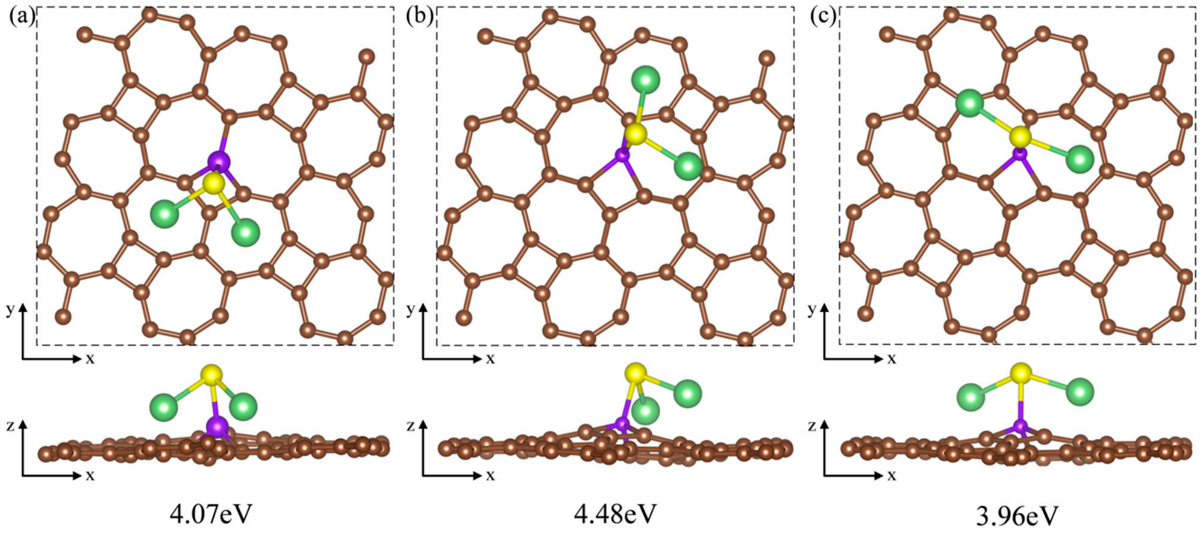

Figure S7. Top and side views of the adsorption configurations and adsorption energies of Li_2S adsorbed on P_{C1}-thgraphene (**a**–**c**).

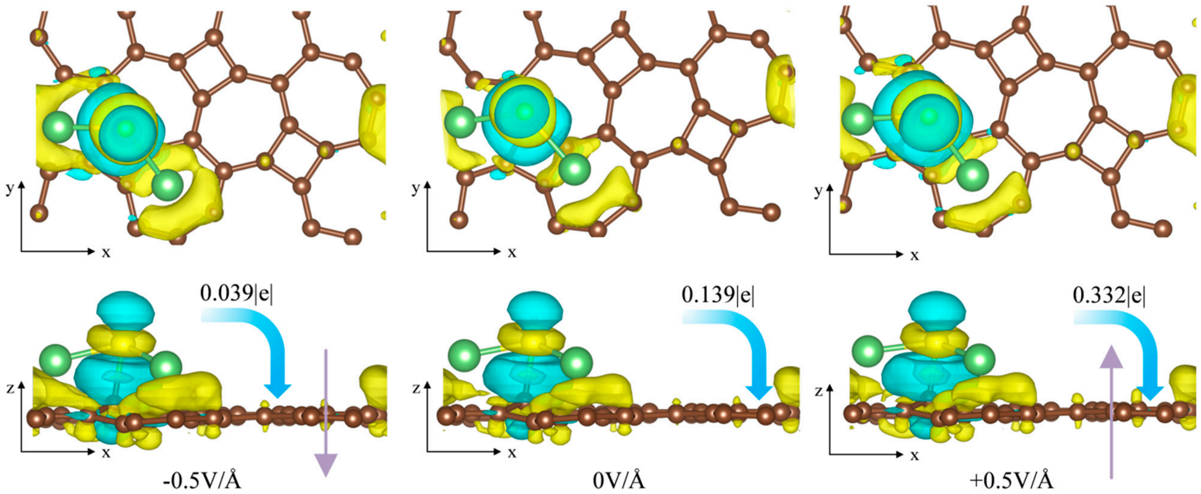

Figure S8. Differential charge density of pristine thgraphene/Li_2S under the external electronic field and the isosurface level is set to be 0.0035 eÅ^{-3}. The light blue region represents electron depletion and the yellow region represents electron accumulation.

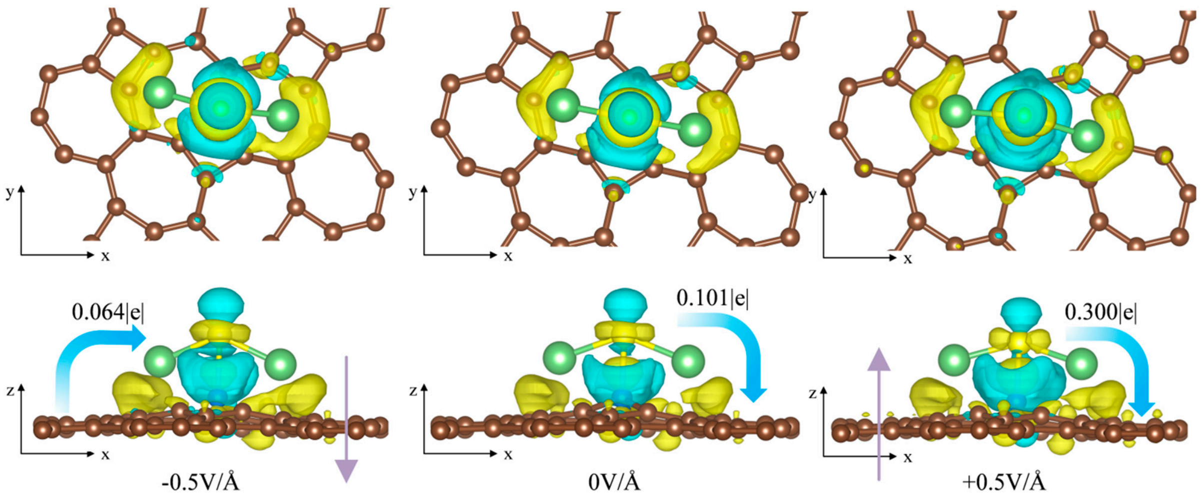

Figure S9. Differential charge density of B_{C1}-thgraphene/Li_2S under the external electronic field and the isosurface level is set to be 0.0035 eÅ^{-3}. The light blue region represents electron depletion and the yellow region represents electron accumulation.

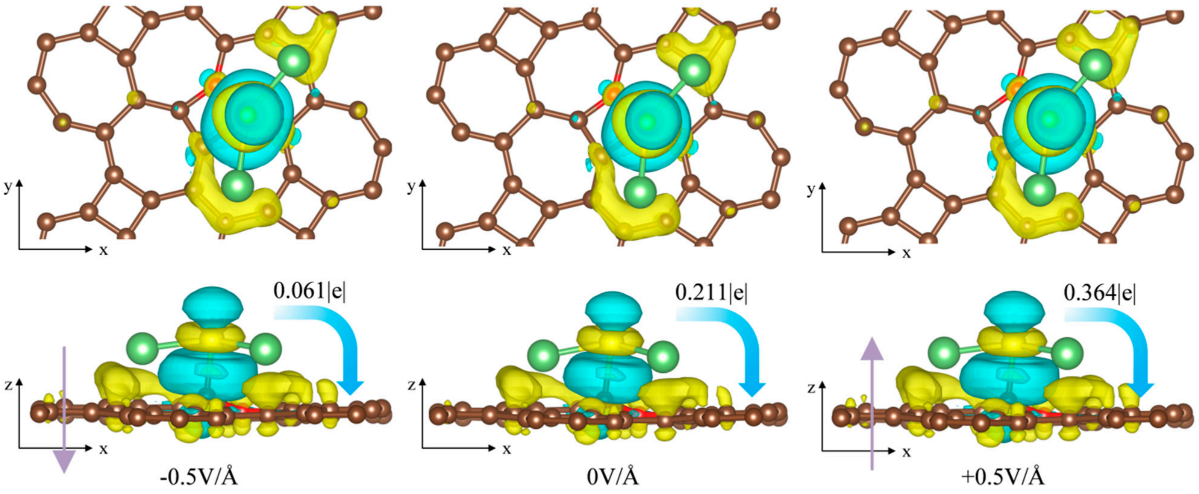

Figure S10. Differential charge density of N_{C1}-thgraphene/Li_2S under the external electronic field and the isosurface level is set to be 0.0035 eÅ^{-3}. The light blue region represents electron depletion and the yellow region represents electron accumulation.

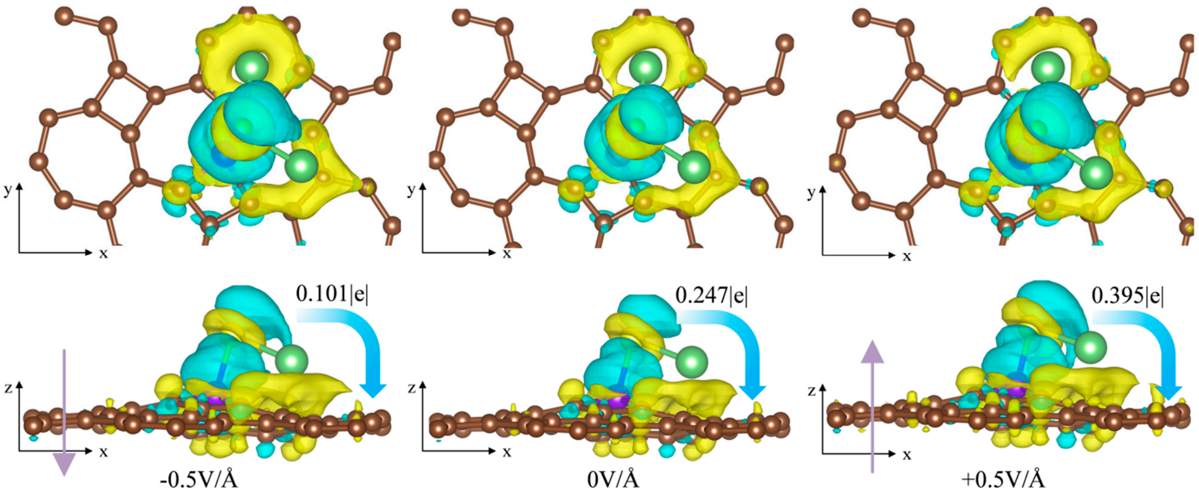

Figure S11. Differential charge density of P_{C1}-thgraphene/Li_2S under the external electronic field and the isosurface level is set to be 0.0035 eÅ^{-3}. The light blue region represents electron depletion and the yellow region represents electron accumulation.

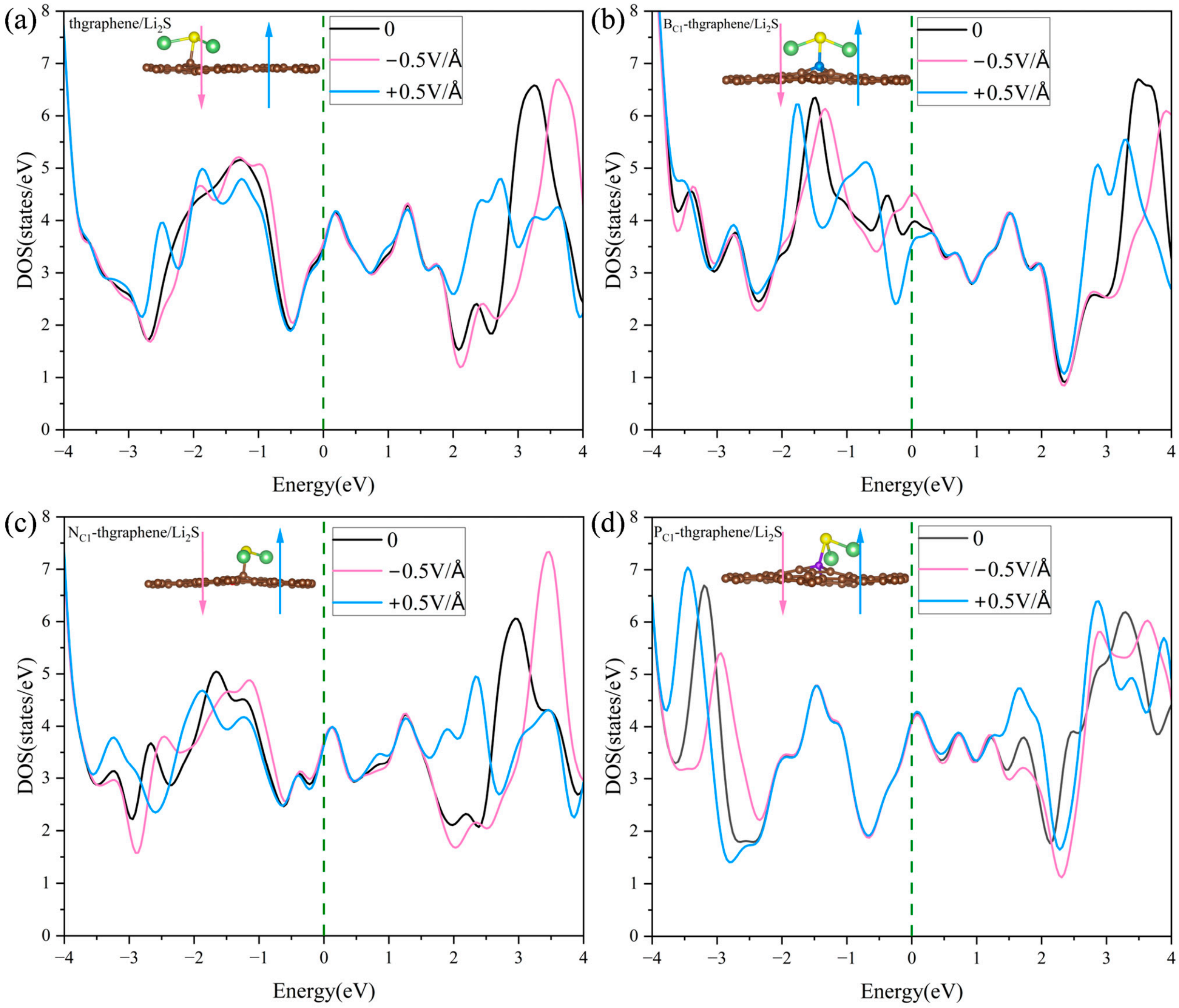

Figure S12. Density of states of Li_2S absorbed on **(a)** thgraphene, **(b)** B_{C1}-thgraphene, **(c)** N_{C1}-thgraphene, **(d)** P_{C1}-thgraphene under the external electric field. The Fermi level is marked by the dark green dashed line.
